# Characterization of the Phytochemical Profile of Halophytes (*Limonium* Mill., Plumbaginaceae) with Natural Deep Eutectic Solvents Extraction

**DOI:** 10.3390/plants14172609

**Published:** 2025-08-22

**Authors:** Antonio Ruiz-Medina, David J. Parras-Guijarro, Carlos Salazar-Mendías, Eulogio J. Llorent-Martínez

**Affiliations:** 1 Department of Physical and Analytical Chemistry, Faculty of Experimental Sciences, University of Jaén, Campus Las Lagunillas, 23071 Jaén, Spain; anruiz@ujaen.es (A.R.-M.); djpguija@ujaen.es (D.J.P.-G.); 2 Department of Animal Biology, Plant Biology and Ecology, Faculty of Experimental Sciences, University of Jaén, Campus Las Lagunillas, 23071 Jaén, Spain; csalazar@ujaen.es

**Keywords:** *Limonium*, phytochemicals, halophyte, HPLC-MS, deep eutectic solvents, green chemistry, environmentally friendly solvents

## Abstract

*Limonium* Mill. (Plumbaginaceae) is a genus comprising many species, some of which are reported to possess high bioactivity and are used as food, medicinal herbs, and fodder. Here we report the use of different deep eutectic solvents (DESs) and natural DESs (NADESs) to study the phytochemical composition of plants of the genus *Limonium* Mill. Most of the organic solvents commonly used for extracting phytochemicals from plants are hazardous to health and may harm the environment. Hence, their replacement with environmentally friendly solvents, particularly NADESs, is desirable. We performed ultrasound-assisted extractions of aerial parts of *Limonium* species using these solvents, followed by characterization of the phytochemicals with liquid chromatography with high-resolution mass spectrometry. Gallic acid and myricetin derivatives accounted for approximately 60 and 27% of all the compounds, respectively, therefore representing more than 80% of the characterized compounds in the analyzed plants. The best extraction yield for both flavonoids and organic acids was achieved using the NADES chloride choline/ethylene glycol (ratio 1:3), improving the recoveries by approximately 20% compared to the use of methanol and methanol/water mixtures. These results indicate the possibility of replacing conventional organic solvents with more environmentally friendly ones, reducing the use of toxic solvents and improving the sample treatment. In addition, possible new *Limonium* plant species were studied in the south of Spain with the optimized method.

## 1. Introduction

Halophyte plants are species that thrive in habitats with high saline concentration, hence representing a sustainable alternative to conventional crops in arid and semi-arid areas, as well as saline soils. *Limonium* Mill., most of whose species are halophytes, is a genus that belongs to the family Plumbaginaceae. It is a megadiverse genus mainly distributed in the Western Mediterranean area (Europe and Africa) [1], inhabiting coastal cliffs, coastal and inland salt marshes, and gypsum outcrops. Most of the *Limonium* species are exclusive to small and isolated areas, many of them being extremely narrow endemics. Due to the specificity of their habitat and the disturbances they are subjected to, a high number of species are considered threatened plants.

*Limonium* species have been reported to possess important bioactivity [2,3,4,5,6] mainly due to their contents of phenolic acids and flavonoids. Hence, some species are used as medicinal herbs [2,3], as well as edible plants in diets for weight reduction, food complement, and fodder [7]. As a result, obtaining information regarding their chemical composition, particularly the content of bioactive compounds, may lead to the development of new management plans for these plant species, as well as future uses as sources of bioactive compounds, primarily phenolic compounds (PCs), for the development of medicinal, food, and feed products.

PCs form the largest class of phytochemicals found in nearly all plants as secondary metabolites. These compounds have been thoroughly investigated due to their potential to prevent a wide range of diseases associated with oxidative stress, as they are natural antioxidants that exhibit significant biological properties, including anti-inflammatory, anticarcinogenic, and antimicrobial effects [8]. A key step for PC analysis in plants is sample treatment, usually performed by solid-phase extraction with different methodologies (ultrasound-assisted extraction, microwave-assisted extraction, etc.), normally using organic solvents such as acetone, ethyl acetate, and alcohols (methanol, ethanol, and propanol) [9,10]. However, from an environmental perspective, these solvents are incompatible with the principles of green chemistry or the sustainability intended in the 2030 Agenda of the United Nations, particularly Sustainable Development Goal 12 (Responsible Consumption and Production). As a result, there is growing interest in developing alternative solvents and more sustainable green extraction methods. An interesting possibility is the use of Deep Eutectic Solvents (DESs). These solvents, discovered by Abbott et al. [11], are distinguished by being mixtures of well-defined compositions that result in a unique and significantly lower melting point in the solid–liquid phase diagram compared to the individual components. They can be used in liquid form at room temperature or temperatures below 70 °C in most cases. DESs are composed of a hydrogen bonding acceptor (HBA) and a hydrogen bonding donor (HBD) and form strong interactions with PCs through hydrogen bonding, dipole–dipole interactions, and Van der Waals forces, improving the extraction process [11]. Natural Deep Eutectic Solvents (NADESs), a subtype of DESs with identical characteristics, have been developed by combining primary metabolites from living organisms with bio-renewable materials [12]. Usually, choline chloride quaternary salt is used as the HBA, while amino acids, sugars, alcohols, or carboxylic acids are used as the HBD for the extraction of PCs [13,14,15]. Some examples of these last compounds are glucose, urea, glycerol, lactic acid, and acetic acid. DESs/NADESs offer several advantages, including low cost, thermal chemical stability in water, and minimal toxicity. Moreover, they are biodegradable, non-volatile, and easy to prepare [15,16,17].

Taxonomy in *Limonium* is quite controversial, as it comprises many agamospecies and frequent intermediate forms (hybrids, varieties, etc.), which makes it difficult to give exact and accepted figures on the number of taxa. Around 470 species and subspecies are recognized in the Mediterranean region [18]. In the case of the Iberian Peninsula and Balearic Islands, the number of taxa ranges from 134 [18] to 127 [19]. New species continue to be described, especially in the dry and semiarid territories of the Southeastern Iberian Peninsula. Specifically, in the Andalusia region, 26 species have been recorded to date [20]. Among all of them, *Limonium quesadense* Erben (endemic to Southeastern Spain) has been studied here, as it is considered an endangered species, appearing in different national and regional red books and lists [21,22].

*L. quesadense* was considered to be a local endemic species to the river Guadiana Menor basin until it was subsequently recorded in the Guadalquivir river basin [23,24]. On the contrary, the aforementioned specimens, together with those collected in the Natural Reserve of Laguna Honda (Alcaudete, Jaén) in the last two decades, have been considered to be hybrids between *L. quesadense* and both *L. supinum* (Girard) Pignatti or *L. delicatulum* (Girard) Kuntze [25]. We therefore believe that there is some uncertainty about the identity of the plants from these localities.

In this work, we have synthesized different DESs/NADESs and evaluated their efficiency for the extraction of phenolic compounds from *Limonium* plants, which are known sources of phenolic compounds, but for which this sample treatment has not been explored to date. Then, several *Limonium* species in non-studied locations were analyzed to discuss the differences between them and evaluate if they belong to *L. quesadense* or to a different subspecies. The characterization of phytochemicals was performed by high-performance liquid chromatography with electrospray ionization and a quadrupole-time-of-flight mass spectrometer (HPLC-ESI-Q-TOF-MS), and the results support the use of NADESs in the sample treatment as a replacement for conventional organic solvents.

## 2. Results and Discussion

### 2.1. Phytochemical Profile by HPLC-ESI-Q-TOF-MS

The first step for the selection of the best extraction solvent was to characterize the phenolic compounds found in the obtained extracts. For this study, leaves from two localities (named ALC and PRI; Figure S1) were extracted using MeOH. The identification of the phenolic compounds was performed using analytical standards (citric acid, coumaric acid, gallic acid, sinapic acid, syringic acid, kaempferol, myricetin, quercetin, and rutin), high-resolution mass spectrometry data (accurate mass, ion source fragmentation, and MS/MS fragmentation), the METLIN database, and bibliographic information. The analytical standards were injected individually as external standards, and their retention times, accurate mass (in MS mode), and fragmentation patterns (in MS/MS mode at collision energies of 10, 20, and 40 V) were used to identify the same compounds in the extracts. Regarding derivatives, such as flavonoid glycosides, the MS/MS fragmentation of the aglycones was used to confirm their identity, whereas the observed neutral losses were used to characterize the attached moieties (such as sugars). The retention times, experimental [M-H]^−^, molecular formula, calculated mass error (ppm), and fragment ions are given in Table 1.

The following is a description of the characterization. In all cases, the molecular formulas were double-checked to confirm the identity of the proposed compounds.

Compound **1** was identified as hibiscus acid due to the deprotonated molecular ion at *m*/*z* 189 and base peak at *m*/*z* 127 [26]. Compound **2** corresponded to malic acid based on its molecular formula and base peak at *m*/*z* 115. Compound **3** was identified as citric acid by comparison with an analytical standard.

Compound **5** was identified as gallic acid by comparison with an analytical standard. Several derivatives were also characterized in the different extracts, all of them showing gallic acid at *m*/*z* 169 and its main fragment ion at *m*/*z* 125. Compound **4** suffered the neutral loss of 162 Da (hexoside moiety, with glucose being the most common sugar) to yield gallic acid at *m*/*z* 169, so it was characterized as galloylglucoside. Compounds **7** and **9** suffered neutral losses of 152 Da (galloyl) and 162 Da (hexoside), so they corresponded to isomers of digalloylglucose. Compound **6** was tentatively characterized as a gallic acid derivative, whereas compound **10** was a dimer of gallic acid.

Compound **8**, with [M-H]^−^ at *m*/*z* 305, was characterized as (epi)gallocatechin by using bibliographic information [27]. With an additional 152 Da (galloyl), compounds **12** and **13** were identified as (epi)gallocatechin gallate isomers.

Compound **11**, with a deprotonated molecular ion at *m*/*z* 477, yielded a fragment ion at *m*/*z* 325 by the loss of 152 Da, which corresponded to a loss of a galloyl moiety, further confirmed by the fragment ions at *m*/*z* 169 and 125. In addition, the neutral loss of 156 Da from 325 to 169 was indicative of a shikimate residue. Hence, the compound was characterized as digalloylshikimic acid [28].

Compounds **14** and **16** were identified as the phenolic acids syringic acid and coumaric acid, respectively, by comparison with analytical standards.

Compound **15** suffered the neutral loss of 162 Da (hexoside) to yield sinapic acid at *m*/*z* 223 (comparison with an analytical standard), so it was tentatively characterized as sinapic acid-glucoside, as glucoside is the most common hexoside moiety in phenolic acid and flavonoid glycosides.

Several glycosides of kaempferol, myricetin, and quercetin were identified in the analyzed plants. The aglycones kaempferol, myricetin (compound **30**), and quercetin (compound **35**) presented [M-H]^−^ at *m*/*z* 285, 317, and 301, respectively; both myricetin and quercetin displayed fragment ions at *m/z* 179 and 151. The aglycones were confirmed by the analysis of their analytical standards (comparing MS/MS fragmentation patterns). Compounds **17**, **19**, **20**, **23**, **28**, **31**, and **33** were myricetin derivatives; compounds **21**, **24**, **25**, **29**, **32**, and **34** were quercetin derivatives; and compounds **26** and **27** were kaempferol glycosides. The different attached moieties of the glycosides were characterized by the neutral losses of 86 Da (malonyl), 146 Da (deoxyhexoside), 152 Da (galloyl), 162 Da (hexoside), and 308 Da (rutinoside).

### 2.2. Selection of the Extraction Solvent

PCs include flavonoids (e.g., anthocyanins, flavonols, flavanols, or isoflavones) and non-flavonoids (e.g., phenolic acids, stilbenes, gallotannins, ellagitannins, or lignins). The most common solvents for the extraction of these compounds from plants are MeOH, ethanol, water, and alcohol/water mixtures; among them, MeOH and MeOH:H_2_O are usually employed. Hence, we compared the extraction achieved for each of the identified compounds with different solvents. For each compound, the peak area of the EIC in MS mode at the corresponding deprotonated molecular ion was used. The percentage was calculated by dividing the individual area of each compound by the total sum of areas of all compounds. Three different sets of data will be discussed: the total sum of all organic acids, the total sum of flavonoids, and the total sum of all the compounds (organic acids + flavonoids). Unless mentioned otherwise, the following information corresponds to leaves collected in the locality named PRI (similar data were obtained for ALC); the comparison between the results of both localities will be made in the end.

**Figure 1:**
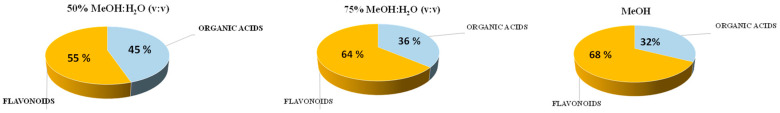
Percentage of organic acids (phenolic + non-phenolic acids) and flavonoids found when using MeOH and MeOH:H_2_O as extraction solvents.

First of all, MeOH and MeOH:H_2_O solvents were tested. We divided the results into two different sets of data, according to the compounds characterized in the extracts: the total sum of flavonoids and the total sum of non-flavonoids. However, considering that only organic acids (mainly phenolic acids) were identified among the non-flavonoids, we will discuss the data considering two main chemical families: flavonoids and organic acids. The results are given in Figure 1; the percentage of non-phenolic acids (Figure S2, Supplementary Material) was very low, so organic acids were mainly composed of phenolic acids. As expected, an increase in MeOH percentage provided a better recovery in flavonoids compared to organic acids due to the polarity of the solvent. However, all the compounds were extracted regardless of the MeOH percentage. In terms of the total area of extracted compounds, similar results were obtained for 75% MeOH and 100% MeOH. Considering the principles of green chemistry, and as a compromise of solvent polarity for organic acids and flavonoids, we selected 75% MeOH as the benchmark solvent in this first step. The relative contribution (%) of each compound is shown in Figure S3 (Supplementary Material). For future discussion, the results obtained with this solvent will be compared with the prepared DESs.

**Figure 2:**
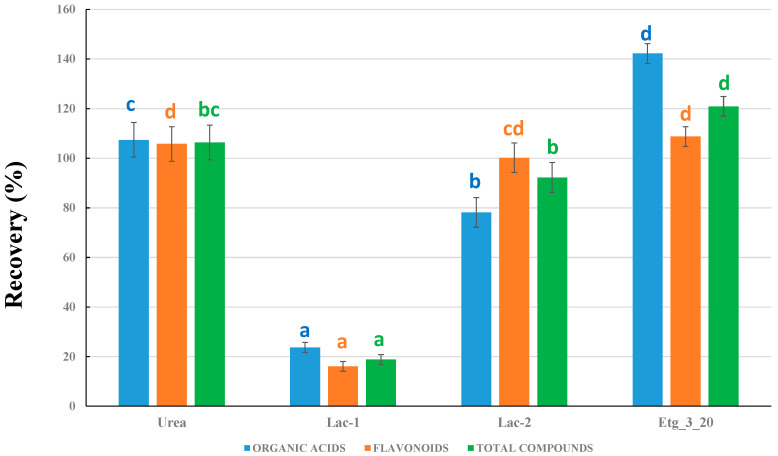
Comparison between the different types of DESs prepared. Recoveries are all referred to 75% MeOH as the benchmark (considered as 100% recovery). Different letters for the columns corresponding to organic acids, flavonoids, or total compounds mean significant differences (*p* ≤ 0.05, n = 3).

In a second experiment, we compared the results obtained for the different DESs prepared in the laboratory (see Table 2 for DES compositions) by using the same percentage of H_2_O in all the DESs, 20% H_2_O, and the same HBA:HBD molar ratio of 1:3 (except for urea, for which 1:2 is the common ratio in the literature). This H_2_O percentage was selected because the common percentages in eutectic solvents are 10 and 20%, and 20% is closer to the 25% H_2_O in the 75% MeOH solvent used as the benchmark. The data in Figure 2 are all referred to the 75% MOH, which was set as a 100% recovery. Four DESs were studied: urea, Lac-1, Lac-2, and Etg_3_20, all of them with a 20% H_2_O content (see Table 2 for nomenclature and composition).

For all solvents, three sets of data are given: flavonoids, organic acids, and the total sum of compounds. It was observed that both choline chloride/urea and choline chloride/ethylene glycol NADESs provided better recoveries than the use of MeOH:H_2_O. In addition, the recoveries obtained with lactic acid were much lower (except for galloylglucose and gallic acid), particularly with sodium acetate (Lac-1). This latter result can be observed in more detail in Figure S4 (Supplementary Material), where the absence of some compounds in both Lac-1 and Lac-2 was observed. As a result, only NADESs based on urea and ethylene glycol were used for further experiments.

Comparing urea and Etg_3_20 NADESs (Figure 2), it can be observed that the improvement in the recovery yields was similar for organic acids and flavonoids for the urea solvent, whereas the use of Etg NADES was particularly useful for organic acids. In addition, the overall results for total compounds were better when using Etg_3_20, which resulted in an improvement of approximately 20% when compared to 75% MeOH. We thus decided to make additional experiments to optimize the nature of the choline chloride/ethylene glycol solvent.

**Figure 3:**
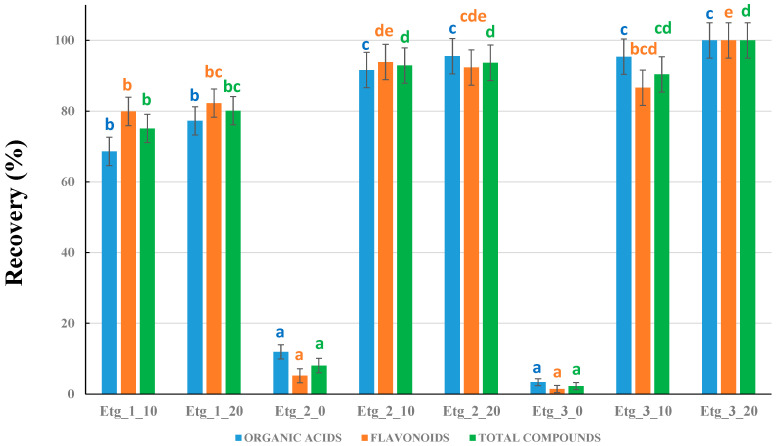
Comparison between the different types of ethylene glycol-based NADESs prepared. Recoveries are all referred to Etg_3_20 as the benchmark (considered as 100% recovery). Different letters for the columns corresponding to organic acids, flavonoids, or total compounds mean significant differences (*p* ≤ 0.05, n = 3).

The last experiment was designed to optimize the composition of the NADES solvents based on choline chloride and ethylene glycol mixtures. Nine different NADESs were intended to be prepared by modifying the HBA:HBD molar ratios, as well as the H_2_O percentages. However, the molar ratio 1:1 in the absence of H_2_O could not be prepared, as a homogeneous liquid was not obtained even when increasing the bathwater temperature and the stirring time. Hence, eight NADESs (Table 2) were prepared and compared. The results are shown in Figure 3. In this case, the benchmark considered as 100% recovery was the best NADES tested so far: Etg_3_20.

First of all, it was observed that at least 10% H_2_O was required in the preparation of NADESs. In the absence of water, the recoveries with both Etg 2_0 and Etg_3_0 were almost negligible. Secondly, the molar ratio 1:1 produced lower recoveries than the other molar ratios (even though 10% and 20% water were used), so Etg_1 was also discarded. We thus performed a more careful comparison between the other NADESs: Etg_2 and Etg_3. Regarding organic acids, there were no significant differences (all data were grouped in ANOVA statistics). Nevertheless, the amount of flavonoids was lower in Etg 3_10, so this NADES can be discarded. For Etg 2_10, 2_20, and 3_20, the results were similar in the statistical analysis. Hence, any of these NADESs could provide satisfactory results at first sight. However, going into more detail, we performed an ANOVA analysis using 75% MeOH, Etg_2_10, Etg_2_20, and Etg_3_20. When 75% MeOH was included, Etg_2_10 presented statistically significant lower recoveries. Therefore, the best NADES solvents were Etg_2_20 and Etg_3_20. Considering that Etg_3_20 provided the highest total area (although no significant differences with Etg_2_20 were observed), we used the data of that NADES for the discussion of the chemical composition of the plants under study.

**Figure 4:**
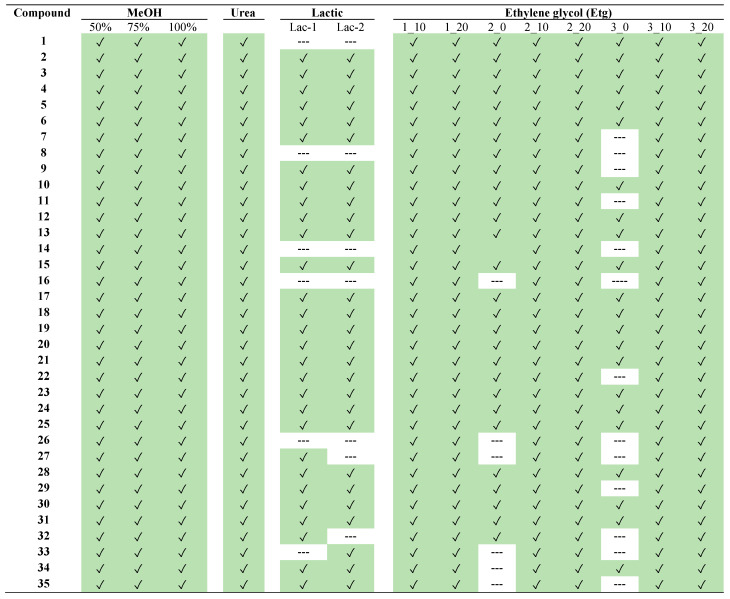
Phytochemicals detected in the different extracts of *Limonium aff. quesadense*. Compounds’ numbers correspond to those indicated in Table 1. Green color is used for the found compounds.

Finally, a summary of the individual compounds found in each extract is depicted in Figure 4. Moreover, the percentages of each compound in all the different extracts of *L. aff. quesadense* are presented in heat maps, using EICs for each compound. The following figures are provided in the Supplementary Materials: Figure S3 for methanolic extracts, Figure S4 for a comparison between DESs based on urea and lactic acid with the optimum Etg DES, and Figure S5 for the characterization of all the Etg DESs. Similarly to the previous discussion, it could be observed that NADESs based on lactic acid, as well as Etg NADESs without water, did not extract some compounds. To sum up, the optimum solvent was found to be the mixture choline chloride-ethylene glycol, with a 20% H_2_O content and a molar ratio of 3:1 (ethylene glycol/choline chloride).

### 2.3. Phytochemical Composition by HPLC-ESI-Q-TOF

After the characterization of the compounds, the composition of ALC and PRI samples was compared with previous results of *L. quesadense* [29]. This comparison was carried out with MeOH, before the solvent optimization, because the results available for *L. quesadense* were obtained using MeOH as a solvent. The comparison, using a heat map, can be seen in Figure S6 (Supplementary Material). The phytochemical profiles were similar from the qualitative point of view, as most of the characterized compounds were the same. However, there were important differences between both plant taxa from the quantitative point of view. The most significant difference was observed for gallic acid (compound **5**), which was a minor compound in *L. quesadense* but one of the main compounds in the plants under study (*L. aff. quesadense*). In addition, the relative contribution of other major compounds (compounds **13**, **23**, **28,** and **29**) was very different. Hence, from the chemical point of view, the results indicate that the plant species located in ALC and PRI localities do not correspond to *L. quesadense*. These results are in agreement with the morphological observations and indicate that the plant species under study is probably a subspecies of *L. quesadense* or even a new species [30].

**Figure 5:**
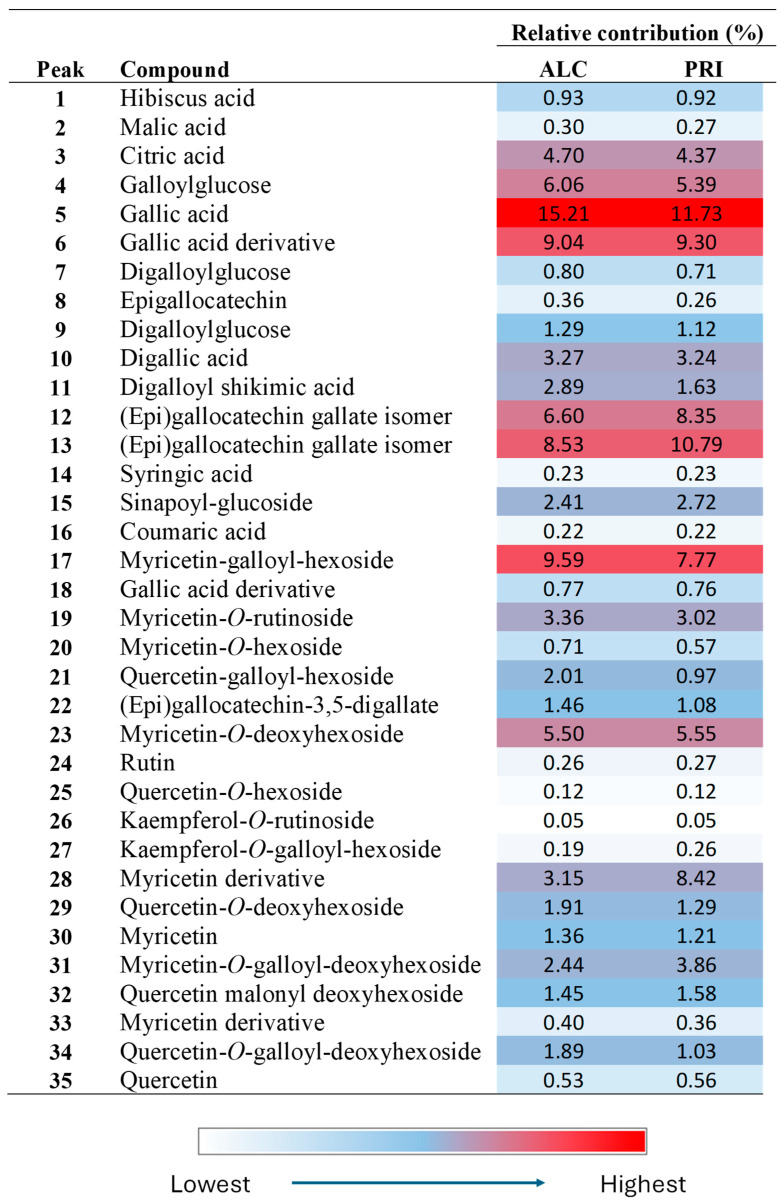
Heat map obtained by HPLC-ESI-Q-TOF in Etg_3_20 extracts of ALC and PRI *Limonium aff. quesadense* samples.

Finally, we will use the data of Etg_3_20 (the best NADES) to discuss the phytochemical composition of the specific species under study. The most abundant compounds in ALC and PRI samples (Figure 5) were gallic acid (compound **5**), as well as gallic acid derivatives, mainly (epi)gallocatechin gallate isomers (compounds **12** and **13**), followed by flavonoids. Among flavonoids, the most important compounds were myricetin derivatives, mainly compounds **17**, **23**, and **28**. Gallic acid and myricetin derivatives accounted for approximately 60 and 27% of all the compounds, respectively. Therefore, more than 80% of the characterized compounds were gallic acid or myricetin derivatives. These results are similar to those found in other species of *Limonium* [29]. Some minor compounds here characterized were hibiscus acid (not reported previously in *Limonium* species to our best knowledge), malic and citric acids, the phenolic acids coumaric, sinapic, and syringic, and several quercetin glycosides. A comparison between the results observed in both localities indicated that their phytochemical contents were similar, hence suggesting that the plants analyzed in both places belong to the same species (results supported by morphological, phytogeographical, and ecological observations). However, the results clearly indicate that the phenolic composition of the analyzed plants does not correspond to *L. quesadense*.

## 3. Materials and Methods

### 3.1. Sample Material

In this work, we have analyzed samples collected in locations not explored so far and more than 90 km away from the classical locality of *L. quesadense*, aiming to assess if these species are *L. quesadense* or a different taxon according to the chemical composition. These plant species were thus named *L. aff. quesadense*. Leaves were collected at two localities from Andalusia (south of Spain) in July 2023 (photographs are provided in Figure S1, Supplementary Material): approximately 20 plant individuals were sampled in each locality, and all the leaves were combined into a sample pool.

- ALC: collected at Alcaudete, Laguna Honda (province of Jaén, Andalusia): 37°35′53.1″ N 4°08′30.7″ W, 448 m a.s.l.
- PRI: collected at Priego de Córdoba, Barranco Cueva de la Reina (province of Córdoba, Andalusia): 37°33′02.9″ N 4°09′08.5″ W, 444 m a.s.l.

Testimonial specimens were stored at the Herbarium of the University of Jaén (code JAEN according to *Index Herbariorum*) [31]. We compared the results obtained for the mentioned samples with those of *L. quesadense*, previously collected at the Native Flora Garden of the University of Jaén, whose phytochemical composition was recently analyzed by our research group [29]. All samples were identified by botanist Dr. Carlos Salazar-Mendías.

### 3.2. Preparation of the Eutectic Solvents

The DESs were prepared by heating, with continuous stirring in a water bath, at 70 °C until a homogeneous colorless liquid was obtained; the DESs were normally formed in under 30 min. The molar ratio of the HBA and HBD components, as well as the water percentage, are both critical parameters that affect the extraction yield, and are shown in Table 2; the checked values are the common ones used in published literature. As the HBA:HBD molar ratio of DESs increases (generally from 1:1 to 1:3), the extraction efficiency also significantly increases. Nevertheless, higher values result in higher viscosity, limiting the mass transfer and decreasing the extraction yield.

Choline chloride was used for all DESs/NADESs, except for lactic acid, for which sodium acetate was also tested. For choline chloride/ethylene glycol NADESs, the nomenclature used was Etg_x_y, where x is the molar ratio of ethylene glycol/choline chloride (HBD:HBA) and y is the water percentage. The DESs remained stable at room temperature after preparation.

DESs are highly viscous due to extensive hydrogen bonding interactions between the components. Their viscosity decreases with the increase in water content, which also produces an improvement in PC extraction yields [32]. Nevertheless, a high and significant increase in water content can weaken their original DES nanostructure and disintegrate it, as well as cause adverse effects on the solution. In the proposed study, 10% and 20% (w/w) of water were added to evaluate the results. It is worth mentioning that the mixture of choline chloride with ethylene glycol could not form a DES in the absence of water.

### 3.3. Sample Preparation

First, leaves were lyophilized (ModulyoD-23, Thermo Savant; Waltham, MA, USA) and ground into powder with a mechanical grinder. Samples were extracted using methanol (MeOH), 75% MeOH:H_2_O (*v*:*v*), 50% MeOH:H_2_O (*v*:*v*), and different DESs (Table 2). The extraction was performed in a single step. For the extraction of the phytochemicals, 0.25 g of the sample was extracted with 5 mL of each solvent by an ultrasonic liquid processor (Qsonica Sonicator; Newtown, CT, USA; power of 55 W and frequency of 20 kHz) at 30% power for 10 min; these conditions were previously optimized in our laboratory. Then, the samples were centrifuged in polypropylene tubes using a high-speed centrifuge (Avanti J-30I, Beckman Coulter, Inc., Indianapolis, Indiana, USA) at 17,500 rpm (35,000× *g*) for 30 min. Finally, the supernatants were filtered (0.45 µm) before HPLC analysis.

### 3.4. Phytochemical Analysis by HPLC-ESI-Q-TOF-MS

The characterization of phytochemicals was performed by HPLC-ESI-Q-TOF-MS. For the analysis of samples, the supernatants obtained in sample preparation (Section 2.3) were directly analyzed—in triplicate—after filtration. An Agilent 1200 (Agilent Technologies, Santa Clara, CA, USA) equipped with an Agilent 6530B Q-TOF MS was used. The analyses were performed with a Luna Omega Polar C_18_ column of 150 × 3.0 mm (5 µm particle size) with a Polar C18 Security Guard cartridge of 4 × 3.0 mm (Phenomenex, Torrance, CA, USA).

The HPLC gradient elution program was carried out with mobile phases of water + formic acid 0.1% *v*/*v* (eluent A) and acetonitrile (eluent B). The gradient program was as follows: 10–25% B in 0–25 min, 25% B in 25–30 min, 25–50% B in 30–40 min, 50–100% B in 40–42 min, and 100% B (42–47 min); then, eluent B was returned to 10% with a 7 min stabilization time. The flow rate was 0.4 mL min^−1^, an injection volume of 10 µL was used, and analyses were performed at room temperature. The parameters used were capillary voltage, 3500 V; nebulizer pressure of 45 psi; drying gas flow rate, 10 L/min; gas temperature, 325 °C; skimmer voltage, 60 V; and fragmentor voltage, 140 V. Data were acquired in negative ion mode using an orthogonal ESI source. The MS and auto MS/MS modes (collision energies of 10, 20, and 40 V) were set to acquire *m*/*z* values ranging between 50 and 1200 at a scan rate of 2 and 3 spectra per second, respectively. Agilent Mass Hunter Qualitative Analysis software version B.06.00 was used for post-acquisition data processing. Molecular formulas, errors (ppm), and fragment ions were obtained by the tool Generate Formulas using the Auto MS/MS algorithm.

For the quantitative discussion, Extracted Ion Chromatograms (EICs) in MS mode were obtained for each compound at its deprotonated molecular ion (using a symmetric expansion of ±5 ppm), and integrated peak areas were used to compare phytochemical profiles between leaves, as well as the extraction efficiency of the different solvents. Relative standard deviations were lower than 5% in all cases.

### 3.5. Statistical Analysis

All experiments were performed in triplicate. Statistical analysis was performed using IBM^®^ SPSS^®^ Statistics software (version 27.0.1.0). A significance level of *p* < 0.05 was applied to one-way ANOVA, followed by Tukey’s post hoc test to identify statistically significant differences between results.

## 4. Conclusions

The interest in DESs/NADESs for PC extraction from plant materials is increasing, and their application represents a promising advancement in green chemistry, addressing both environmental concerns and efficiency in the extraction process. The novelty of this research lies in utilizing DESs/NADESs to enhance the extraction efficiency and yield of bioactive compounds from *Limonium* plants compared to conventional extraction with MeOH or EtOH. An improvement in extraction yields of approximately 20% was achieved with choline chloride/ethylene glycol NADES when compared to the conventional use of MeOH, hence providing a low-cost, environmentally friendly extraction method.

After the method was optimized, the phytochemical composition of the leaves of *L. aff. quesadense* collected from two localities was characterized. Data revealed an abundance of gallic acid derivatives and myricetin glycosides as the main compounds. The chemical profiles of the plants collected in both localities were similar, hence indicating they belong to the same species. However, the compositions were different from the quantitative point of view from the chemical profile of *L. quesadense* Erben, hence concluding that the analyzed species is probably a subspecies of *L. quesadense* or even a new species. These results indicate that this approach may be useful to evaluate the identity of halophyte species in non-studied locations by just comparing the chemical profile of the main phenolic compounds.

## Figures and Tables

**Table 1:** Characterization of the compounds in the methanolic extracts of *Limonium aff. quesadense*.

No.	t*_R_* (min)	Observed [M-H]^−^	Molecular Formula	Error (ppm)	Fragment Ions	Assigned Identification
**1**	1.9	189.0042	C_6_H_6_O_7_	−1.39	127.0032	Hibiscus acid
**2**	2.0	133.014	C_4_H_6_O_5_	1.84	115.0041, 71.0145	Malic acid
**3**	2.4	191.0194	C_6_H_8_O_7_	1.79	173.0078, 129.0179, 111.0083, 87.0090	Citric acid *
**4**	2.5	331.0676	C_13_H_16_O_10_	−1.44	271.0453, 169.0134, 125.0243	Galloylglucose
**5**	3.1	169.0141	C_7_H_6_O_5_	0.97	124.9837, 114.9884	Gallic acid *
**6**	4.5	325.0562	C_14_H_14_O_9_	−0.46	169.0142, 125.0254	Gallic acid derivative
**7**	6.7	483.0791	C_20_H_20_O_14_	−2.51	331.0692, 271.0455, 169.0124, 125.0242	Digalloylglucose
**8**	7.5	305.0662	C_15_H_14_O_7_	1.05	219.0662, 179.0364, 125.0234	Epigallocatechin
**9**	9.5	483.0786	C_20_H_20_O_14_	−1.24	331.0659, 169.0135, 125.0248	Digalloylglucose
**10**	10.2	321.0255	C_14_H_10_O_9_	−1.02	169.0138, 125.0241	Digallic acid
**11**	11.7	477.0674	C_21_H_18_O_13_	−0.78	325.0557, 169.0150, 125.0227	Digalloyl shikimic acid
**12**	12.4	457.0779	C_22_H_18_O_11_	−0.53	331.0450, 305.0665, 169.0138, 125.0245	(Epi)gallocatechin gallate isomer
**13**	13.2	457.0774	C_22_H_18_O_11_	0.59	331.0457, 305.0661, 287.0568, 193.0143, 169.0138, 125.0242	(Epi)gallocatechin gallate isomer
**14**	14.2	197.0457	C_9_H_10_O_5_	−0.72	124.0161, 78.0113	Syringic acid *
**15**	14.2	385.1137	C_17_H_22_O_10_	0.87	223.0819, 179.0361, 137.0226	Sinapoyl-glucoside
**16**	15.6	163.0399	C_9_H_8_O_3_	−0.06	119.0505	Coumaric acid *
**17**	15.7	631.0938	C_28_H_24_O_17_	0.40	479.0819, 316.0225, 271.0310	Myricetin-galloyl-hexoside
**18**	17.4	477.1045	C_22_H_22_O_12_	−1.52	433.1144, 401.5665, 313.0572, 169.0135, 125.0238	Gallic acid derivative
**19**	17.5	625.1409	C_27_H_30_O_17_	0.17	317.0318, 316.0195, 271.0197, 178.9963, 151.0035	Myricetin-*O*-rutinoside
**20**	17.9	479.0834	C_21_H_20_O_13_	−0.60	316.0234, 271.0250, 179.0025, 151.0035	Myricetin-*O*-hexoside
**21**	19.7	615.0990	C_28_H_24_O_16_	0.40	463.0900, 301.0369, 178.9980, 151.0037	Quercetin-galloyl-hexoside
**22**	19.9	609.0887	C_29_H_22_O_15_	0.17	457.0747, 169.0139, 125.0248	(Epi)gallocatechin-3,5-digallate
**23**	20.5	463.0880	C_21_H_20_O_12_	0.52	316.0219, 178.9978	Myricetin-*O*-deoxyhexoside
**24**	20.6	609.1445	C_27_H_30_O_16_	2.59	301.0354, 169.0132	Rutin *
**25**	21.1	463.0876	C_21_H_20_O_12_	1.79	301.0342, 179.2098	Quercetin-*O*-hexoside
**26**	23.4	593.1507	C_27_H_30_O_15_	1.63	285.0398, 257.0334, 151.0052	Kaempferol-*O*-rutinoside
**27**	23.9	599.1029	C_28_H_24_O_15_	1.31	447.0903, 313.0575, 285.0477, 169.0152	Kaempferol-*O*-galloyl-hexoside
**28**	24.3	549.0885	C_24_H_22_O_15_	0.37	505.0976, 316.0222, 271.0241, 178.9983, 151.0031	Myricetin derivative
**29**	24.9	447.0928	C_21_H_20_O_11_	1.27	301.0843, 179.0015, 151.0032	Quercetin-*O*-deoxyhexoside
**30**	26.8	317.0308	C_15_H_10_O_8_	−1.72	179.9897, 151.0026	Myricetin *
**31**	28.4	615.0988	C_28_H_24_O_16_	0.48	463.0879, 317.0294, 179.0006, 151.0024	Myricetin-*O*-galloyl-deoxyhexoside
**32**	29.6	533.0933	C_24_H_22_O_14_	0.22	447.1043, 301.0332	Quercetin malonyl deoxyhexoside
**33**	31.6	701.1001	C_31_H_26_O_19_	−0.87	549.0885, 505.0992, 463.0971, 316.0229, 271.0266, 178.9981	Myricetin derivative
**34**	33.2	599.1034	C_28_H_24_O_15_	1.52	447.0959, 301.0351, 178.9961, 151.0067	Quercetin-*O*-galloyl-deoxyhexoside
**35**	33.9	301.0353	C_15_H_10_O_7_	0.05	179.0104, 151.0051	Quercetin *

* Identified by comparison with the analytical standard. Hexoside means glucoside or galactoside; deoxyhexoside means rhamnoside or furanoside.

**Table 2:** Nomenclature and information of the prepared DESs.

Abbreviation	HBA	HBD	Water (%)	Mol Ratio
Urea	Choline chloride	Urea	20	1:2
Lac-1	Sodium acetate	Lactic acid	20	1:3
Lac-2	Choline chloride	Lactic acid	20	1:3
Etg_1_10	Choline chloride	Ethylene glycol	10	1:1
Etg_1_20	Choline chloride	Ethylene glycol	20	1:1
Etg_2_0	Choline chloride	Ethylene glycol	0	1:2
Etg_2_10	Choline chloride	Ethylene glycol	10	1:2
Etg_2_20	Choline chloride	Ethylene glycol	20	1:2
Etg_3_0	Choline chloride	Ethylene glycol	0	1:3
Etg_3_10	Choline chloride	Ethylene glycol	10	1:3
Etg_3_20	Choline chloride	Ethylene glycol	20	1:3

## Data Availability

The data presented in this study are available in the present article. The original contributions presented in this study are included in the article/Supplementary Materials. Further inquiries can be directed to the corresponding author.

## Supplementary Materials

The following supporting information can be downloaded at: https://www.mdpi.com/article/10.3390/plants14172609/s1, Figure S1: Photographs of *Limonium aff. quesadense*. Upper left: ALC locality; upper right: PRI locality; bottom left: vegetative phase; bottom right: flowering phase; Figure S2. Percentage of flavonoids and organic acids in the methanolic extracts of *Limonium aff. quesadense*; Figure S3. Heat map obtained by HPLC-ESI-Q-TOF in the methanolic extracts of *Limonium aff. quesadense* (n = 3); Figure S4. Heat map obtained by HPLC-ESI-Q-TOF in the urea, Lac-1, Lac-2 and Etg_3_20 extracts of *Limonium aff. quesadense* (n = 3); Figure S5. Heat map obtained by HPLC-ESI-Q-TOF in all the Etg DES extracts of *Limonium aff. quesadense* (n = 3); Figure S6. Heat map obtained by HPLC-ESI-Q-TOF in the methanolic extracts of *Limonium aff. quesadense* and *L. quesadense*. The results in the first column are the average of ALC and PRI samples.
