# Supplementary material for: Characterization of the Phytochemical Profile of Halophytes (Limonium Mill., Plumbaginaceae) with Natural Deep Eutectic Solvents Extraction
Source: Plants (Basel). 2025 Aug 22;14(17):2609. doi: 10.3390/plants14172609 (PMC12430231; doi:10.3390/plants14172609)
Supplement: Supplementary file 1 [file plants-14-02609-s001.zip › plants-3828777-supplementary.pdf]

Characterization of the Phytochemical Profile of Halophytes (*Limonium* Mill., Plumbaginaceae) with Natural Deep Eutectic Solvents Extraction

Antonio Ruiz-Medina [1], David J. Parras-Guijarro [1], Carlos Salazar-Mendías [2] and Eulogio J. Llorent-Martínez [1,*]

[1] Department of Physical and Analytical Chemistry, Faculty of Experimental Sciences, University of Jaén, Campus Las Lagunillas, 23071 Jaén, Spain; anruiz@ujaen.es (A.R.-M.); djpguija@ujaen.es (D.J.P.-G.)
[2] Department of Animal Biology, Plant Biology and Ecology, Faculty of Experimental Sciences, University of Jaén, Campus Las Lagunillas, 23071 Jaén, Spain; csalazar@ujaen.es
* Correspondence: ellorent@ujaen.es

Figure S1. Photographs of *Limonium aff. quesadense*. Upper left: ALC locality; upper right: PRI locality; bottom left: vegetative phase; bottom right: flowering phase.

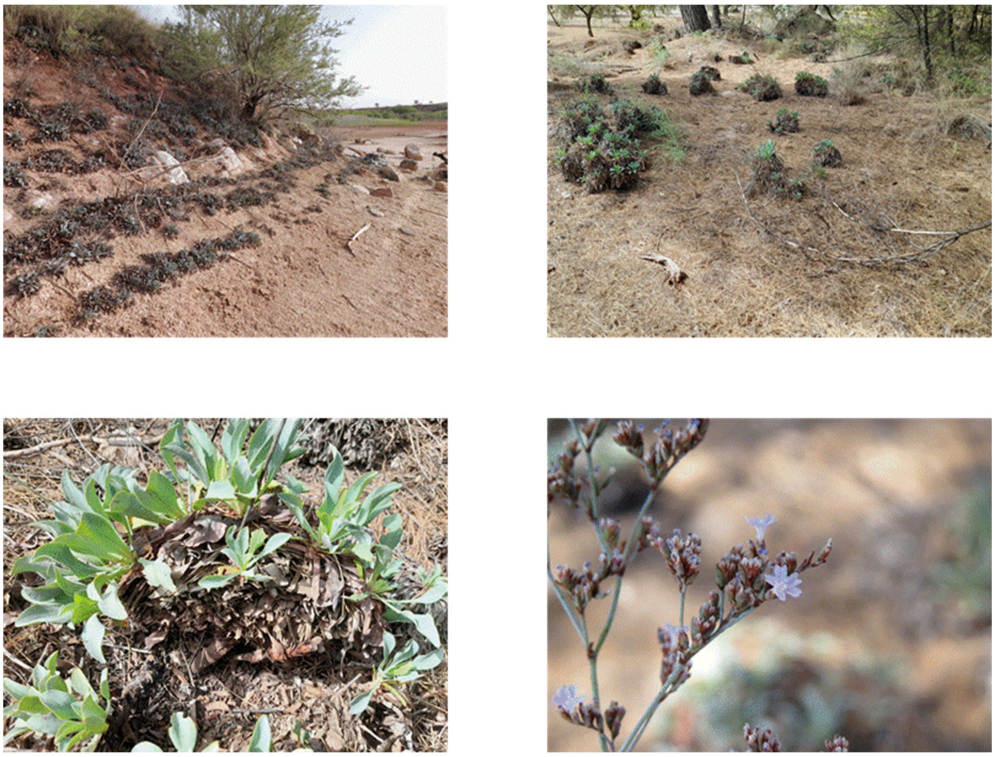

ALC: collected at Alcaudete, Laguna Honda (province of Jaén, Andalusia): 37°35'53.1"N 4°08'30.7"W, 448 m a.s.l. PRI: collected at Priego de Córdoba, Barranco Cueva de la Reina (province of Córdoba, Andalusia): 37°33'02.9"N 4°09'08.5"W, 444 m a.s.l.

Figure S2. Percentage of flavonoids and organic acids in the methanolic extracts of *Limonium aff. quesadense*.

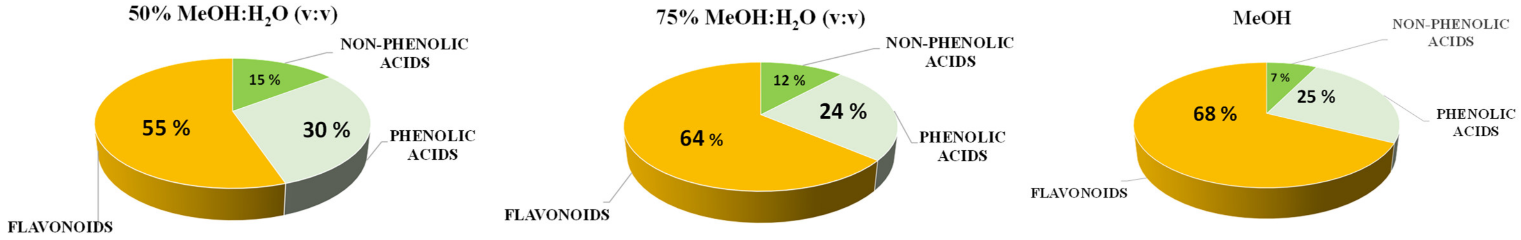

Figure S3. Heat map obtained by HPLC-ESI-Q-TOF in the methanolic extracts of *Limonium aff. quesadense* (n=3).

		Relative contribution (%)		
**Peak**	**Compound**	**50% MeOH**	**75% MeOH**	**MeOH**
1	Hibiscus acid	7.18	6.31	5.29
2	Malic acid	0.56	0.42	0.36
3	Citric acid	6.96	5.09	1.66
4	Galloylglucose	4.36	1.13	2.98
5	Gallic acid	11.01	10.82	11.28
6	Gallic acid derivative	4.01	1.97	2.79
7	Digalloylglucose	1.69	0.30	0.20
8	Epigallocatechin	0.26	0.17	0.19
9	Digalloylglucose	1.02	0.58	0.42
10	Digallic acid	3.34	2.62	1.97
11	Digalloyl shikimic acid	1.36	0.63	0.48
12	(Epi)gallocatechin gallate isomer	9.06	5.67	5.48
13	(Epi)gallocatechin gallate isomer	7.94	10.35	9.21
14	Syringic acid	0.02	0.02	0.01
15	Sinapoyl-glucoside	2.53	2.19	1.46
16	Coumaric acid	0.24	0.27	0.38
17	Myricetin-galloyl-hexoside	5.75	5.69	4.12
18	Gallic acid derivative	0.52	3.86	3.16
19	Myricetin-*O*-rutinoside	2.81	2.74	3.31
20	Myricetin-*O*-hexoside	0.58	0.77	0.66
21	Quercetin-galloyl-hexoside	0.92	1.04	0.90
22	(Epi)gallocatechin-3,5-digallate	1.28	2.82	1.30
23	Myricetin-*O*-deoxyhexoside	5.50	6.56	9.35
24	Rutin	0.35	1.37	0.60
25	Quercetin-*O*-hexoside	0.16	0.18	0.28
26	Kaempferol-*O*-rutinoside	0.09	0.19	0.07
27	Kaempferol-*O*-galloyl-hexoside	0.29	0.35	0.36
28	Myricetin derivative	10.03	11.54	14.57
29	Quercetin-*O*-deoxyhexoside	1.45	1.95	2.44
30	Myricetin	0.73	1.34	1.93
31	Myricetin-*O*-galloyl-deoxyhexoside	4.20	6.35	7.66
32	Quercetin malonyl deoxyhexoside	1.97	2.20	2.41
33	Myricetin derivative	0.54	0.63	0.60
34	Quercetin-*O*-galloyl-deoxyhexoside	0.96	1.27	1.34
35	Quercetin	0.32	0.62	0.79

Lowest → Highest

Figure S4. Heat map obtained by HPLC-ESI-Q-TOF in the urea, Lac-1, Lac-2 and Etg_3_20 extracts of *Limonium aff. quesadense* (n=3).

		Relative contribution (%)			
**Peak**	**Compound**	**Urea**	**Lac-1**	**Lac-2**	**Etg 3_20**
1	Hibiscus acid	0.50	0.00	0.00	0.92
2	Malic acid	0.04	0.28	0.05	0.27
3	Citric acid	5.22	1.00	2.85	4.37
4	Galloylglucose	2.98	9.82	0.66	5.39
5	Gallic acid	12.36	22.54	15.72	11.73
6	Gallic acid derivative	5.61	4.94	3.24	9.30
7	Digalloylglucose	0.41	0.47	1.44	0.71
8	Epigallocatechin	0.39	0.00	0.00	0.26
9	Digalloylglucose	1.00	0.36	0.16	1.12
10	Digallic acid	2.42	2.30	0.93	3.24
11	Digalloyl shikimic acid	0.29	1.16	1.86	1.63
12	(Epi)gallocatechin gallate isomer	7.82	5.73	10.56	8.35
13	(Epi)gallocatechin gallate isomer	9.83	13.01	13.86	10.79
14	Syringic acid	0.19	0.00	0.00	0.23
15	Sinapoyl-glucoside	2.62	1.79	2.54	2.72
16	Coumaric acid	0.30	0.00	0.00	0.22
17	Myricetin-galloyl-hexoside	8.12	3.89	6.62	7.77
18	Gallic acid derivative	2.63	0.97	1.22	0.76
19	Myricetin-*O*-rutinoside	5.03	2.87	3.67	3.02
20	Myricetin-*O*-hexoside	1.64	0.78	0.41	0.57
21	Quercetin-galloyl-hexoside	1.97	1.00	1.06	0.97
22	(Epi)gallocatechin-3,5-digallate	1.07	1.40	2.46	1.08
23	Myricetin-*O*-deoxyhexoside	3.67	8.64	7.63	5.55
24	Rutin	0.63	0.73	0.71	0.27
25	Quercetin-*O*-hexoside	0.16	0.18	0.13	0.12
26	Kaempferol-*O*-rutinoside	0.05	0.00	0.00	0.05
27	Kaempferol-*O*-galloyl-hexoside	0.35	0.33	0.00	0.26
28	Myricetin derivative	10.09	8.49	10.53	8.42
29	Quercetin-*O*-deoxyhexoside	1.49	1.23	1.23	1.29
30	Myricetin	1.54	1.15	2.80	1.21
31	Myricetin-*O*-galloyl-deoxyhexoside	4.86	3.20	4.92	3.86
32	Quercetin malonyl deoxyhexoside	2.00	0.75	0.00	1.58
33	Myricetin derivative	0.28	0.00	0.40	0.36
34	Quercetin-*O*-galloyl-deoxyhexoside	1.55	0.45	1.14	1.03
35	Quercetin	0.91	0.56	1.21	0.56

Lowest → Highest

Figure S5. Heat map obtained by HPLC-ESI-Q-TOF in all the Etg DES extracts of *Limonium aff. quesadense* (n=3).

		**Relative contribution (%)**							
**Peak**	**Compound**	Etg_1_10	Etg_1_20	Etg_2_0	Etg_2_10	Etg_2_20	Etg_3_0	Etg_3_10	Etg_3_20
1	Hibiscus acid	0.55	0.82	17.16	0.72	0.76	19.92	1.01	0.92
2	Malic acid	0.16	0.26	2.73	0.22	0.23	3.61	0.32	0.27
3	Citric acid	2.65	4.71	8.51	3.86	5.22	12.27	4.18	4.37
4	Galloylglucose	4.74	5.15	6.70	4.79	4.40	4.57	5.23	5.39
5	Gallic acid	14.46	13.55	21.06	13.46	13.25	17.59	13.26	11.73
6	Gallic acid derivative	6.94	7.06	3.02	8.70	8.43	2.31	9.70	9.30
7	Digalloylglucose	0.62	0.48	0.19	0.54	0.52	0.00	0.69	0.71
8	Epigallocatechin	0.36	0.42	0.20	0.28	0.47	0.00	0.32	0.26
9	Digalloylglucose	1.05	0.92	0.30	0.98	0.95	0.00	1.19	1.12
10	Digallic acid	2.69	2.92	0.84	2.78	3.05	0.77	3.29	3.24
11	Digalloyl shikimic acid	1.44	1.48	0.40	1.60	1.55	0.45	1.86	1.63
12	(Epi)gallocatechin gallate isomer	10.03	10.73	4.36	7.93	8.04	3.53	9.05	8.35
13	(Epi)gallocatechin gallate isomer	9.78	9.66	12.73	11.39	11.34	12.39	12.23	10.79
14	Syringic acid	0.06	0.12	0.26	0.86	1.84	0.00	0.18	0.23
15	Sinapoyl-glucoside	2.58	2.51	1.26	2.53	2.30	1.31	3.04	2.72
16	Coumaric acid	0.25	0.25	0.00	0.22	0.22	0.00	0.26	0.22
17	Myricetin-galloyl-hexoside	6.46	6.36	2.36	7.25	6.99	2.37	8.37	7.77
18	Gallic acid derivative	0.75	0.85	0.74	0.76	0.74	0.73	0.76	0.76
19	Myricetin-*O*-rutinoside	3.34	3.00	2.18	3.15	2.89	2.19	3.30	3.02
20	Myricetin-*O*-hexoside	0.62	0.57	0.75	0.55	0.52	0.78	0.63	0.57
21	Quercetin-galloyl-hexoside	1.03	0.92	0.60	0.99	0.87	0.58	1.09	0.97

22	(Epi)gallocatechin-3,5-digallate	1.05	0.97	0.47	1.05	1.07	1.12	1.07	1.08
23	Myricetin-*O*-deoxyhexoside	6.18	5.78	5.71	5.69	5.39	5.66	6.09	5.55
24	Rutin	0.35	0.27	0.23	0.42	0.41	0.26	0.30	0.27
25	Quercetin-*O*-hexoside	0.14	0.18	0.10	0.13	0.12	0.13	0.13	0.12
26	Kaempferol-*O*-rutinoside	0.08	0.06	0.00	0.05	0.05	0.00	0.05	0.05
27	Kaempferol-*O*-galloyl-hexoside	0.31	0.27	0.19	0.27	0.25	0.00	0.29	0.26
28	Myricetin derivative	10.13	9.49	3.45	8.58	8.56	3.59	0.93	8.42
29	Quercetin-*O*-deoxyhexoside	1.39	1.30	0.87	1.28	1.21	0.88	1.43	1.29
30	Myricetin	1.39	1.25	0.53	1.29	1.17	0.59	1.36	1.21
31	Myricetin-*O*-galloyl-deoxyhexoside	4.71	4.23	1.76	4.13	3.93	1.80	4.46	3.86
32	Quercetin malonyl deoxyhexoside	1.64	1.60	0.33	1.55	1.47	0.37	1.70	1.58
33	Myricetin derivative	0.34	0.33	0.00	0.33	0.32	0.00	0.41	0.36
34	Quercetin-*O*-galloyl-deoxyhexoside	1.09	0.99	0.00	1.08	0.94	0.25	1.15	1.03
35	Quercetin	0.63	0.53	0.00	0.59	0.54	0.00	0.66	0.56

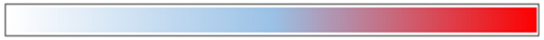

Lowest → Highest

Figure S6. Heat map obtained by HPLC-ESI-Q-TOF in the methanolic extracts of *Limonium aff. quesadense* and *L. quesadense*. The results in the first column are the average of ALC and PRI samples.

		Relative contribution (%)	
**Peak**	**Compound**	***L. aff. quesadense***	***L. quesadense***
1	Hibiscus acid	5.48	0.12
2	Malic acid	0.39	0.00
3	Citric acid	1.78	0.58
4	Galloylglucose	3.27	0.43
5	Gallic acid	13.40	0.14
6	Gallic acid derivative	2.83	0.39
7	Digalloylglucose	0.22	0.00
8	Epigallocatechin	0.24	1.09
9	Digalloylglucose	0.47	0.00
10	Digallic acid	2.04	0.00
11	Digalloyl shikimic acid	0.69	0.00
12	(Epi)gallocatechin gallate isomer	5.03	1.64
13	(Epi)gallocatechin gallate isomer	8.46	17.18
14	Syringic acid	0.01	0.00
15	Sinapoyl-glucoside	1.41	1.32
16	Coumaric acid	0.40	0.00
17	Myricetin-galloyl-hexoside	4.76	6.92
18	Gallic acid derivative	3.28	1.52
19	Myricetin-*O*-rutinoside	3.60	6.24
20	Myricetin-*O*-hexoside	0.76	2.20
21	Quercetin-galloyl-hexoside	1.43	1.59
22	(Epi)gallocatechin-3,5-digallate	1.58	0.65
23	Myricetin-*O*-deoxyhexoside	9.59	35.28
24	Rutin	0.61	0.26
25	Quercetin-*O*-hexoside	0.28	0.00
26	Kaempferol-*O*-rutinoside	0.07	0.07
27	Kaempferol-*O*-galloyl-hexoside	0.32	0.64
28	Myricetin derivative	10.18	5.33
29	Quercetin-*O*-deoxyhexoside	3.14	10.96
30	Myricetin	2.12	0.15
31	Myricetin-*O*-galloyl-deoxyhexoside	6.40	4.09
32	Quercetin malonyl deoxyhexoside	2.38	0.50
33	Myricetin derivative	0.65	0.00
34	Quercetin-*O*-galloyl-deoxyhexoside	1.97	0.73
35	Quercetin	0.79	0.00

Lowest → Highest
